# Happy 100th, structural biology

**DOI:** 10.1063/4.0000788

**Published:** 2025-11-18

**Authors:** George N. Phillips, Eaton Edward Lattman

**Affiliations:** 1 Department of BioSciences, Rice University, Houston, Texas 77005, USA; 2 Department of Materials Design and Innovation, University at Buffalo, Buffalo, New York 14260, USA

## Abstract

About 100 years ago, the field of structural biology was born, led by James B. Sumner who recognized that enzymes were molecules with specific functions. In its contemporary form structural biology is used to interpret and understand molecular and cellular function, to design drugs, and to advance biotechnology in general.

The period from 1925 to 1930 was one of astonishing productivity and creativity in science, and we are now marking the centenaries of many of these achievements. It encompasses the birth of modern quantum mechanics, beginning with de Broglie's suggestion that the wave particle duality that was already being used in the description of light also applied to particles other than the photon, particles with mass.^1^ In 1925, Heisenberg proposed a matrix formulation of quantum mechanics,^2^ for which an English translation appears in Ref. 3. This was closely followed by Schrödinger's wave equation approach^4,5^ with Born clarifying the physical meaning of the wave function.^6^ This culminated in Heisenberg's uncertainty principle that formalized the impossibility of knowing simultaneously the values of certain pairs of observables of a particle, such as the position and momentum.

Other transformative advances of that era include Fleming's discovery of penicillin,^7^ Hubble's observation of the expanding universe,^8^ and Payne's revelation that stars are primarily composed of hydrogen and helium.^9^

Less heralded but equally foundational was the birth of what we now call structural biology. See Ref. 10 for an expansive history of structural and molecular biology. In its contemporary form, structural biology involves the determination of the three-dimensional atomic structure of macromolecules such as proteins, and the use of these structures to understand molecular and cellular function, to design drugs, and to advance biotechnology in general. The field has been associated with many Nobel prizes. The term “structural biology” came into common use in the 1980s, but its intellectual history extends much further back in time. In this review we suggest, with the benefit of hindsight, that birth of structural biology occurred 100 years ago, with two critical experiments published in 1926. One was Sumner's crystallization of the enzyme urease^11^ showing that enzymes are proteins with uniform atomic structures. Urease was known by chemical tests to be a protein, and the extremely high specific activity of dissolved urease crystals demonstrated that the enzymatic activity was coincident with this protein. Enzymes were proteins, and the molecules of these proteins could indeed form crystals, thus demonstrating that they adopt uniform atomic structures.

The other was Svedberg's analytical ultracentrifugation^12^ of hemoglobin, establishing its molecular weight as ∼66 000 Daltons,^13^ corroborating earlier estimates based on iron content. Further, the detailed shape of the trace of concentration vs radius curve measured in the centrifuge cell strongly suggested that that the sample was monodisperse and did not display an equilibrium among species with molecular weights that were various multiples of 16 700. The protein hemoglobin was a molecule within the existing meaning of the term, and it had a known molecular weight. Svedberg and Nichols soon reported similar findings for egg albumin.^14^

Sumner's achievement came despite personal adversity—he lost an arm in an accident—and professional skepticism. At the time, Willstätter and colleagues argued that enzymes could not be proteins.^15^ But Sumner's paper lists seven distinct reasons why he believed that his octahedral crystals of a jack bean globulin were identical to urease. To the modern ear, these points seem clear and compelling, a fresh reminder of how paradigm shifts alter viewpoints. In the event, Sumner published an additional 10 papers on urease, gradually increasing the weight of the evidence. But it took fully a decade, and the accumulation of much work by other groups, for the concept that enzymes were proteins to take hold. Notably, his original paper never used the word “molecule.”

In 1926, just before the experiments described above, there was still confusion about the nature of proteins. They were known through the work of Emil Fischer to comprise amino acids linked together by peptide bonds. At the same time the physical form of proteins was believed to be colloidal. Colloids are dispersions of small particles in a carrier substance. The suspensions are typically cloudy, indicating that the particles are too large to dissolve like small molecules, but too small settle out. Gelatin is an example from that period. The arrangement of amino acids within proteins was unclear. But the idea that a particular protein could have many conformations had been discussed.

Further confounding the situation was confusion about the nature of enzymes. They were operationally defined as biological preparations that had the capacity to catalyze chemical actions with great specificity. Fischer's lock-and-key model^16^ provided a helpful analogy, but the “lock”—the enzyme—remained a mystery.

It is thus readily apparent that the joint fields of enzymology and protein science were poised for transformational thinking. It seems clear looking back that the work of Sumner and of Svedberg flipped the conceptual switch, and helped transform protein and enzymology research. Shortly afterward the enzyme pepsin was also crystallized, and later pepsin crystals were shown to yield x-ray diffraction patterns, suggesting to the imaginative mind of Bernal^17^ that the determination of the structure of proteins by crystallography might one day be possible.

Protein structure determination advanced hierarchically. Fischer's work and Edman's sequencing method^18^ confirmed that each protein has a unique “primary” structure. But how did these amino acid chains form crystals?

As early as the 1930s, Astbury observed repeating patterns—“alpha” and “beta”—in fiber x-ray diffraction of proteins.^19–21^ Pauling, Corey, and Branson proposed alpha-helices,^22^ and Crick showed how helical structures explain diffraction patterns.^23^ Pauling and Corey refined the beta-sheet model in 1951,^24^ establishing the concept of “secondary” structure.

The full 3D structure of proteins, however, remained elusive due to the “phase problem.” At the MRC in Cambridge, Kendrew and Perutz overcame this challenge, revealing the structures of myoglobin^25^ and hemoglobin,^26^ both heme proteins.

They expected orderly alpha-helices but discovered complex, unexpected folds. Hemoglobin, revealed as a tetramer of two different polypeptide chains, expanded the molecular concept to include non-covalent assemblies—“quaternary” structure. This work laid the foundation for understanding protein folding and evolution.

X-ray crystallography has since yielded thousands of macromolecular structures, cataloged in the PDB. Structural biology of nucleic acids began with Watson and Crick's DNA model,^27^ based on Franklin's diffraction images^28^ and Chargaff's rules.^29^ Like protein structural biology, nucleic acid structure has been crucial for understanding life and enabling biotechnology.

Recent advances include cryo-electron microscopy, now capable of near-atomic resolution for massive molecular assemblies such as viruses and bacterial motors.^30^ Structure prediction from sequence has made enormous strides,^31^ and researchers increasingly focus on molecular dynamics—how structures move and change in function.

Structural biology's century of progress has deepened our understanding of life's processes and enabled breakthroughs in medicine and biotechnology.

## Data Availability

Data sharing is not applicable to this article as no new data were created or analyzed in this study.
